# Comparative Evaluation of ^68^Ga-Citrate PET/CT and ^18^F-FDG PET/CT in the Diagnosis of Type II Collagen-Induced Arthritis in Rats

**DOI:** 10.1155/2019/2353658

**Published:** 2019-03-19

**Authors:** Zi Wang, Liang Cai, Tingting Xu, Dan Tang, Lin Liu, Yue Chen

**Affiliations:** ^1^Department of Nuclear Medicine, Affiliated Hospital of Southwest Medical University, Nuclear Medicine and Molecular Imaging Key Laboratory of Sichuan Province, Luzhou, Sichuan, China; ^2^State Key Laboratory for Quality Research of Chinese Medicines, Macau University of Science and Technology, Taipa, Macau; ^3^Department of Pathology, Affiliated Hospital of Southwest Medical University, Luzhou, Sichuan, China

## Abstract

Rheumatoid arthritis (RA) is a chronic autoimmune disease characterized by systemic, symmetrical, and erosive synovitis. RA is one of the most common disabling diseases in the clinic. The main clinical intervention strategies are early diagnosis and early treatment. This study aims to predict the diagnostic value of ^68^Ga-citrate and ^18^F-FDG PET/CT in RA by comparing and analyzing the value of ^68^Ga-citrate and ^18^F-FDG PET/CT for diagnosing type II collagen-induced arthritis (CIA) in rats. Some CIA models were established. Normal rats were selected as the control group, and 23 days and 40 days were selected as the early and late time points of arthritis, respectively. The semiquantitative analysis of CIA rats was carried out with ^68^Ga-citrate PET/CT and ^18^F-FDG PET/CT, and the ratio of the maximum standardized uptake (SUV_max_) values in the regions of interest (ROIs) of the hind foot ankle joint and thigh muscle was calculated and statistically analyzed. The distribution of CIA rats in vivo at the ^68^Ga-citrate 90 min time point was studied, and the ankle tissues were evaluated with hematoxylin and eosin (HE) staining. ^68^Ga-citrate PET/CT is obviously superior to ^18^F-FDG PET/CT for CIA imaging, and the statistical results show that the difference between the two examination methods is statistically significant (*P* < 0.001). The uptake of these two radiopharmaceuticals showed the same trend in arthritis rats with different scores. The distribution of ^68^Ga-citrate at 90 min is consistent with the trend shown by ^68^Ga-citrate PET/CT. ^68^Ga-citrate PET/CT can reflect the inflammatory activity of affected joints in CIA rats earlier and more sensitively than ^18^F-FDG PET/CT, and this imaging advantage continues until the later stage of inflammation. Therefore, ^68^Ga-citrate PET/CT is worthy of further promotion and application in the clinical diagnosis of RA.

## 1. Introduction

Rheumatoid arthritis (RA) is a chronic autoimmune disease characterized by systemic, symmetrical, and aggressive synovitis [1]. RA has an insidious onset and is recurrent and persistent. In patients with RA, the affected joints show redness, heat, and pain in the early stages of the disease. In the late stages of the disease, there are different degrees of joint deformity and associated atrophy of bones and muscles. Irreversible joint damage eventually leads to disability. RA is one of the most common disabling diseases in the clinic. The etiology and pathogenesis of RA are not fully understood and may be related to genetic, environmental, and immune abnormalities. The clinical intervention strategy for RA is early diagnosis and treatment. The timely and accurate diagnosis of RA is of great clinical significance for relieving the clinical symptoms of the disease and reducing the disability rate.

Laboratory evaluation of RA patients involves several biological parameters, including rheumatoid factor, C-reactive protein (CRP), matrix metalloproteinase-3 (MMP-3), and erythrocyte sedimentation rate (ESR) [2]. However, these biological parameters do not accurately reflect the activity of the disease. Magnetic resonance imaging (MRI) and ultrasound have been used to detect synovitis early in RA [3, 4]. Unfortunately, MRI is often limited to a single joint, and the use of MRI for total body joint imaging is impractical; furthermore, ultrasound is largely dependent on the physician's experience.

Bone scintigraphy is also a routine imaging method used to evaluate RA, but this modality is not sensitive enough to detect synovitis [5]. Positron emission tomography/computed tomography (PET/CT) has higher resolution and can better distinguish bone and soft tissue structures than other imaging modalities. ^18^F-FDG PET/CT can accurately reflect glucose metabolism levels in organs and tissues. This imaging modality has become a powerful imaging technology recognized in oncology. This imaging technology can also be used to evaluate inflammatory diseases, such as RA [6, 7].

^67^Ga-citrate is a classic radiopharmaceutical for inflammatory imaging and has been widely used in the clinic [8]. ^68^Ga is an isotope of ^67^Ga and has similar physical and chemical properties to ^67^Ga. As a positron nuclide, ^68^Ga can be combined with PET/CT technology to obtain high-resolution images. The half-life of ^68^Ga (68 min) is significantly lower than that of ^67^Ga (78.3 h), so ^68^Ga-labeled compounds can significantly reduce the radiation dose to patients and staff. In addition, ^68^Ga is obtained by a ^68^Ge/^68^Ga generator [9], which is convenient and quick to obtain, and the cost is obviously lower than that of ^67^Ga and ^18^F produced by an accelerator. Therefore, PET/CT imaging with ^68^Ga-labeled citrate has considerable social and economic benefits. This paper aims to compare the value of ^68^Ga-citrate and ^18^F-FDG PET/CT for evaluating collagen-induced arthritis (CIA) in rats.

## 2. Materials and Methods

### 2.1. Materials

An itG ^68^Ge/^68^Ga generator was purchased from China Isotope and Radiation Corporation. ^18^F-FDG was produced and synthesized by the Department of Nuclear Medicine, Affiliated Hospital of Southwest Medical University; its radiochemical purity (RCP) was over 99%. High-purity chick type II collagen was purchased from Chondrex, USA, and incomplete Freund's adjuvant was purchased from Sigma, USA. Isoflurane was purchased from Hebei Yipin Pharmaceutical Co., Ltd., China, and its chemical formula is C3H2CIF5O. All other chemicals involved are reagent-grade materials purchased from Aladdin Bio-Chem Technology, Shanghai, China. Xinhua No. 1 chromatography paper used for paper chromatography (PC) was purchased from Hangzhou Xinhua Paper Co., Ltd. Rat ankle joint data acquisition was performed with a micro-PET/CT scanner (SIEMENS InveonTM, Munich, Germany). The radioactivity of the samples was measured using a *γ* counter (SN-695B; Hesuo Rihuan Photoelectric Instrument Co., Shanghai, China) and a calibrator (CRC-15R; Capintec Inc., Florham Park, NJ, USA). Seven-week-old healthy and clean female Sprague-Dawley (SD) rats were provided by the Animal Experimental Center of Southwestern Medical University (Animal License SCXK 2013–17) and weighed 147 g ± 12 g.

### 2.2. Animal Models

The CIA model has been widely used as a model for human RA because of its common pathological and immunological characteristics. The specific operation for establishing CIA rat models is as follows: 10 mg of chick type II collagen fiber was dissolved in 5 ml of 0.1 mol/L acetic acid and mixed evenly so that the concentration of the mixed solution is 2 mg/ml. This mixture was then stored at 4°C for one night. The next day, 0.7 ml of collagen fiber was aspirated with a 2 ml syringe, and 0.7 ml of incomplete Freund's adjuvant was aspirated with another 2 ml syringe. The needles were removed from both syringes taken, at the same time, the three-way stopcock was connected to an ice bag, and the knob of the three-way stopcock was adjusted to allow the two syringes to communicate at a right angle; thus, the remaining interface was closed. The syringes were pushed back and forth on the ice bag for 30 min so that the solutions in the two syringes were fully mixed into a milky white substance. If the resultant emulsion cannot be dispersed when immersed into clear water, the preparation of 1 mg/ml collagen emulsion can be considered complete. The prepared female SD rats were anesthetized with isoflurane gas (3% concentration, oxygen flow rate 0.6 L/min), and the anesthesia was considered successful when the muscular tension of the rats disappeared. The collagen emulsion (0.1 ml) was injected subcutaneously at a distance of 2-3 cm from the tail root of the rats, and a total of 12 rats were immunized. One week after the initial immunization, the same dose of collagen emulsion was injected into the same area in these rats again.

Beginning on the day of the second immunization, rats were examined clinically every other day to assess the severity of paw arthritis. The arthritis index (AI) score of the paw of model rats is as follows: score 0 = no macroscopic evidence of erythema and swelling; score 1 = erythema and mild soft tissue swelling limited to the tarsus/ankle joint; score 2 = erythema and mild soft tissue swelling extending from the ankle joint to the tarsus; score 3 = erythema and moderate soft tissue swelling extending from the ankle joint to the tarsus; score 4 = obvious erythema and severe swelling (including ankle joint, plantar surface, and toe) or ankylosis of the limbs.

### 2.3. Preparation and Quality Control of ^68^Ga-Citrate

Preparation of ^68^Ga-citrate: A ^68^Ge/^68^Ga generator with a specification of 20 mCi was rinsed with 0.05 M HCI to yield 5 ml of ^68^Ga^3+^ solution for radioactive labeling. During rinsing, the ^68^Ga^3+^ solution was split into 5 EP (2 ml) tubes in sequence so that each EP tube contained 1 ml of solution. The third tube with the highest activity (about 7 mCi ^68^Ga^3+^) was obtained and 43 mg of citrate solid was added, followed by agitation and reaction in a 60°C water bath for 10–15 min. The pH was adjusted to 4-5, and the solution was filtered through a 0.22 *μ*m filter membrane to obtain the final solution. The quality of ^68^Ga-citrate and ^68^Ga^3+^ solutions was determined with a PC bar. The origin was marked 2 cm from one end of Xinhua No. 1 paper with a pencil, and 2–4 *µ*l of the final solution was applied at the origin. Finally, a mixture of NH_4_OH (25.28%)/CH_3_OH (100%)/H_2_O (1 : 5 : 9.5 V/V/V) was used as the elution solvent, and TLC technology was used to analyze the radiochemical purity of ^68^Ga-citrate.

### 2.4. Stability Assessment of ^68^Ga-Citrate

To evaluate the in vitro stability of ^68^Ga-citrate, TLC was used to analyze its quality at room temperature (22 ± 3°C) at 30 min, 60 min, 90 min, 120 min, and 180 min after the final preparation was synthesized. To study the stability of the labeled preparation in human plasma, 0.1 ml of fresh healthy adult plasma was taken, approximately 300 *µ*Ci (approximately 0.08 ml) ^68^Ga-citrate was added, and the radiochemical purity of the labeled compound was determined by TLC after incubation at 37.4°C for 30 min, 60 min, 90 min, 120 min, and 180 min.

### 2.5. Micro-PET/CT Imaging of CIA Rats and Normal Rats

To evaluate the optimal imaging point for CIA rats injected with ^68^Ga-citrate, we selected two affected rats (scoring 1–3), anesthetized them with isoflurane gas (3% concentration, oxygen flow rate 0.6 L/min), weighed them rapidly, and then injected 0.1 ml of 150–300 *µ*Ci ^68^Ga-citrate in isotonic saline through the tail vein. Under continuous gas anesthesia (1.5% concentration, oxygen flow rate 0.5 L/min), the animals were placed in the supine position for fixation. Micro-PET/CT was performed on the posterior bilateral ankle joints 10 min, 30 min, 60 min, 90 min, 120 min, 150 min, 180 min, and 210 min after injection, and the acquisition matrix size was 128 × 128. Each scan lasted for 10 min, and the acquired data were reconstructed as two-dimensional filtered back-projections (2D-FBPs). After the data collection was completed, the region of interest (ROI, mm^3^) of the lateral muscles of the ankle joint and ipsilateral hind leg was delineated with Inveon Research Workplace 4.2 software, and the maximum standardized uptake value (SUV_max_) was calculated to determine the ratio of the arthritis area (target (*T*) area) to background (nontarget (NT) area) (*T*/NT).

Before performing imaging studies on all the arthritis rats, we selected 3 healthy female rats for ^68^Ga-citrate/^18^F-FDG PET/CT imaging of the posterior ankle joint as a control group. These rats were weighed after anesthesia with isoflurane gas, and then 200 *µ*Ci ^68^Ga-citrate/^18^F-FDG in approximately 0.1 ml of isotonic saline was injected via the tail vein. The imaging time point in rats injected with ^68^Ga-citrate was the best time as determined above, while in rats injected with ^18^F-FDG, micro-PET/CT image acquisition was initiated at 60 min [10]. We usually performed ^68^Ga-citrate PET/CT examinations on rats in the morning on one day. After at least 5 ^68^Ga half-lives, these rats were injected with ^18^F-FDG in the same way for imaging.

On the 23rd and 40th days after the first immunization, we collected micro-PET/CT data from the hind ankle joints of CIA model rats. Like the control rats, ^68^Ga-citrate PET/CT examinations were performed on these rats in the morning on one day. After at least 5 half-lives of ^68^Ga, ^18^F-FDG was injected into these rats in the same way for imaging. Before all imaging experiments, rats were fasted, and access to water was restricted. After injecting radiopharmaceuticals, the rats were placed separately in small cages to avoid fighting and excessive exercise.

### 2.6. Biodistribution in the CIA Rats

The biodistribution of ^68^Ga-citrate in vivo at 90 min was evaluated in CIA model rats (weight range 153–176 g). Each rat was injected with 280–300 *µ*Ci of ^68^Ga-citrate in an equal volume of saline via the tail vein in a total volume of 0.1 ml. After 90 minutes, isoflurane (2% concentration, oxygen flow rate of 0.6 L/min) was administered for anesthesia, and 0.1 ml of blood was obtained through the tail vein. Then, 1 ml of air was injected through the tail vein to embolize the rat, and then the rat was quickly dissected. The heart, liver, spleen, lungs, kidneys, stomach, small intestines, muscles, skin, brain, and bilateral hind foot ankle joints of the rats were removed, washed with physiological saline and dried, placed in a test tube of known quality, and a *γ* counter and microbalance were used to measure the radiological counts and quality of these tissues and calculate the percentage of injected dose per gram of tissue (%ID/g).

### 2.7. Histology

After the biological distribution study was completed, the collected ankle tissues were routinely fixed in 10% neutral formalin. Then, these tissues were sent to the Pathology Department of the Affiliated Hospital of Southwest Medical University, where professional researchers completed decalcification, fixation, sectioning, hematoxylin-eosin (HE) staining, and microscopic observation of the pathological tissues of the ankle joints of the arthritis model rats.

### 2.8. Statistical Analysis

The *T*/NT ratio and biological distribution data were reported as $\bar{X}\pm \text{SD}$. The statistical significance of the 95% confidence level was determined by paired *t*-tests using SPSS statistics 17.0 software package, and the significance level was set to 0.001.

## 3. Results

### 3.1. CIA Models

On the 3rd day after the second immunization, one rat was found dead, and the other 11 rats showed no signs of deterioration in diet, defecation, or vitality. On the 15th day after the first immunization, one rat developed slight redness and swelling in the left hind foot ankle joint. As time progressed, the left and right ankle joints of several rats showed redness, swelling, and lameness, and the ankle joints of some rats showed explosive deterioration in 1-2 days. On day 23, 8 rats developed arthritis (AI score ≥ 1). On day 40, 10 rats developed arthritis. A total of 10 of the 11 surviving rats used to establish the CIA model successfully developed arthritis (90.9%).

### 3.2. Synthesis and Stability of ^68^Ga-Citrate

It is simple and convenient to synthesize ^68^Ga-citrate using readily available reagent-grade citrate and ^68^Ga^3+^ solution directly rinsed with our generator. The radiochemical purity and labeling rate were determined by PC and TLC. ^68^Ga^3+^ remains at the origin (Rf = 0), while the labeling compound ^68^Ga-citrate moves to the front (Rf = 1) under the same solvent conditions (NH_4_OH/CH_3_OH/H_2_O). The radiochemical purity of ^68^Ga-citrate has been proven to be over 99%, so it can meet the requirements for subsequent experiments and practical applications. The stability of the labeled compound measured at room temperature and in human plasma at 37.4°C shows that the in vitro labeling rate of ^68^Ga-citrate at 180 min is higher than 97%.

### 3.3. Micro-PET/CT Image Analysis

CIA rats were used to acquire ^68^Ga-citrate images at different time points. The SUV_max_ value of the ankle joint was measured by placing a 3D volume of interest around each paw. The middle lateral muscle of the tibia of rats was selected as the background; the volume of the 3D ROI of approximately 2.5 × 2.5 × 2.5 mm was drawn with analysis software to obtain the SUV_max_. The SUV_max_ was measured at least 5 times, the highest and lowest SUV_max_ were removed, and the remaining values were used to calculate the average value to obtain the final SUV_max_ of the muscle background. Next, the SUV_max_ of the ankle joint was divided by the SUV_max_ of the muscle background for standardization, and finally, the ratio of the *T* area (ankle joint) and NT area (muscle) (*T*/NT) was obtained by calculation. Ten minutes after injection of ^68^Ga-citrate, CIA model rats had light uptake in the bilateral ankle joints. At 30 min, the uptake of the imaging agent increased gradually. At 90–120 min, the ratio of *T*/NT was basically stable, and the ratio did not change significantly with the acquisition time. However, the image quality gradually deteriorated after 150 min.

**Figure 1:**
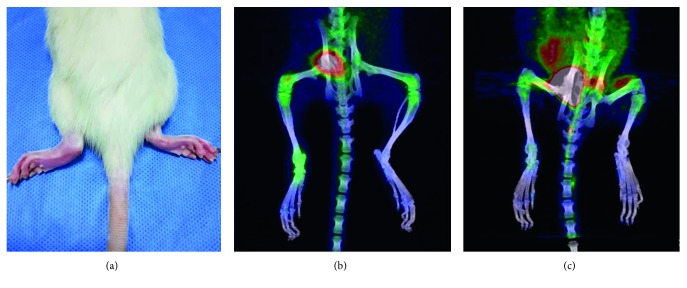
On the 23rd day, the AI score of one rat's left paw was 2 and that of its right paw was 0. On PET/CT images, ^68^Ga-citrate showed high uptake in the left ankle joint (SUV_max_, 2.2) but low uptake in the right ankle joint (SUV_max_, 1). On ^18^F-FDG images, the left paw showed low uptake of the imaging agent (SUV_max_, 1.4), and the right ankle joint also showed low uptake (SUV_max_, 0.96).

CIA onset in rats is similar to that in RA, and the disease process is gradual. The degree of redness and swelling of the bilateral paws of these rats was often inconsistent.  Figure 1 shows that on the 23rd day, the AI score in the left paw of one rat was 2 points, while that in the right paw was 0 points. On PET/CT images, ^68^Ga-citrate showed high uptake in the left ankle joint (SUV_max_, 2.2) but low uptake in the right ankle joint (SUV_max_, 1). In ^18^F-FDG images, the left paw had low uptake of the imaging agent (SUV_max_, 1.4), and the right ankle joint had low uptake (SUV_max_, 0.96).

**Figure 2:**
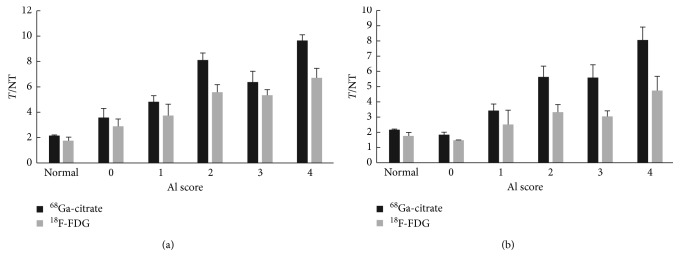
CIA rats were examined with ^68^Ga-citrate PET/CT and ^18^F-FDG PET/CT at 23 (a) and 40 days (b) after immunization to evaluate the ratio of the ankle joint (target area) to the muscle background (nontarget area) (*T*/NT) of these rats. Normal rats were collected in advance as a control group.

The ratio of *T*/NT calculated after the two imaging agents were administered to CIA rats and normal rats were compared (Figure 2). The ratio of ankle joint uptake in rats with successfully induced arthritis (score 1–4) was significantly higher than that in normal rats. At 23 and 40 days, the uptake of the two imaging agents in the ankle joints of arthritic rats followed a general trend: score 4 > score 2 > score 3 > score 1. The *T*/NT ratio of ^68^Ga-citrate was always higher than that of ^18^F-FDG for rats with the same scores on 23 or 40 days. For rats with the same scores, the *T*/NT ratio of the two imaging agents at 23 days was always higher than that at 40 days.

The *T*/NT ratios of these 11 arthritic rats were statistically analyzed (Table 1). On the 23rd day, the *T*/NT ratio for ^68^Ga-citrate imaging (6.34 ± 2.70) in arthritic rats was 1.626 (95% CI: 1.128–2.124), which was higher than that for ^18^F-FDG imaging (4.71 ± 1.77); the difference was statistically significant (*t*(21) = 6.795, *P* < 0.001). On the 40th day, the *T*/NT ratio for ^68^Ga-citrate imaging (5.14 ± 2.10) was 1.992 (95% CI: 1.462–2.522), which was higher than that for ^18^F-FDG imaging (3.15 ± 1.26); the difference was statistically significant (*t*(21) = 7.819, *P* < 0.001). We believe that ^68^Ga-citrate is better than ^18^F-FDG for imaging of CIA rats with micro-PET/CT.

### 3.4. Biodistribution

**Figure 3:**
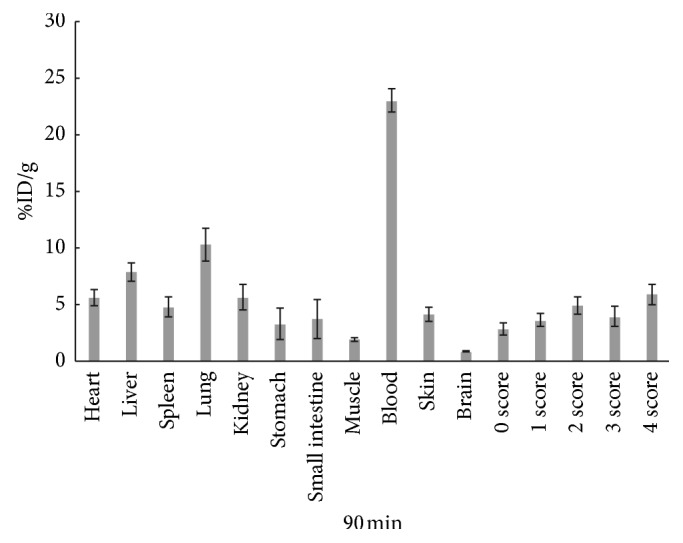
Distribution in vivo of arthritis in rats after injection of ^68^Ga-citrate at 90 min.

The biological distribution of ^68^Ga-citrate in arthritic rats was studied.  Figure 3 summarizes the data of %ID/g at 90 min. The evaluation of arthritic ankles followed a general trend: score 4 (5.88 ± 0.90%ID/g) > score 2 (4.88 ± 0.77%ID/g) > score 3 (3.92 ± 0.86%ID/g) > score 1 (3.61 ± 0.59%ID/g) > score 0 (2.84 ± 0.53%ID/g). These data are consistent with the PET/CT images obtained above: the highest uptake was scored 4, and the lowest uptake was scored 0. Blood uptake was the highest (23.03 ± 1.04%ID/g) 90 min after ^68^Ga-citrate injection. The next highest uptake occurred in the lungs (10.27 ± 1.44%ID/g) and liver (7.89 ± 0.82%ID/g). As the main aggregation site of transferrin, the liver also showed significant uptake. The stomach (3.30 ± 1.40%ID/g), small intestine (3.70 ± 1.73%ID/g), and kidneys (5.62 ± 1.10%ID/g) all also showed uptake, indicating that the tracer could be excreted both through the digestive and urinary tracts.

### 3.5. Histology

**Figure 4:**
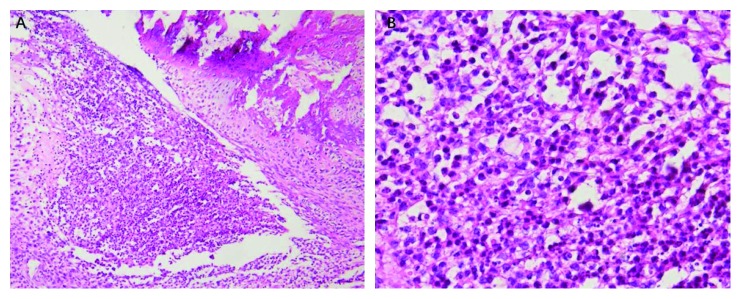
When the magnification is 10 × 10 (a), some serous fluid in the joint cavity can be seen exuding, accompanied by a large number of neutrophils infiltrating the area, which is a manifestation of suppurative inflammation. When magnification is 10 × 40 (b), neutrophils can be seen in the full field of vision, and lymphocytes and plasma cells are hardly visible.

For HE staining of tissues, the ankle tissues of CIA rats fixed in 10% neutral formalin solution were removed, decalcified in 10% formic acid-formaldehyde solution, and dehydrated in 50–100% gradient ethanol. Then, these tissues were immersed in a mixed solution of 100% ethanol and xylene (1 : 1 V/V) and embedded in paraffin after becoming transparent. After embedding, 4 *µ*m paraffin sections were prepared. These slices were dried in a dryer for 30–60 min, dewaxed with xylene for 5–10 min, and hydrated with gradient ethanol (100–75%). Next, HE was used in sequence. Finally, the tissues were dehydrated with gradient ethanol (75–100%), became transparent with the application of xylene, and were sealed with neutral gum. All sections were photographed at 10 × 10 and 10 × 40 magnification.  Figure 4 shows a magnification of 10 × 10 (Figure 4(a)) visible partial exudation of the joint cavity with a large number of neutrophils infiltrating the area, which is an indicative of suppurative inflammation; a magnification of 10 × 40 (Figure 4(b)) shows visible neutrophils in the full field of view, with almost no visible lymphocytes and plasma cells.

## 4. Discussion

The CIA model was Trentham's first experimental arthritis model that was established in 1977 [11]. After a large number of experimental studies, the model has been recognized as a classic model for studying human RA. There are many similarities between pathological changes and symptoms of CIA rats and RA [12]. First, the clinical symptoms are symmetrical joint involvement and invasion of the distal joints of limbs. Second, the main pathological change is proliferative synovitis, which gradually develops from synovitis to pannus formation and begins with edge destruction, eventually leading to articular cartilage destruction and even bone destruction. Finally, CIA causes autoimmunity and is controlled by the major histocompatibility complex genes and related genes. The cellular and humoral immune changes are obvious. However, there are also some differences between CIA and human RA that cannot be ignored. First, the polymorphonuclear cells can be observed in the synovium of CIA rats in the early stage. However, in the early pathological changes of human RA, although the synovium presents obvious hyperemia, edema, and synovial coating cells proliferation accompanied by plasma cells and lymphocyte infiltration, polymorphonuclear cells are abundant in synovial fluid and rare in synovium [13]. Second, CIA disease progresses rapidly and usually reaches the advanced stage within a few months, while human RA disease takes several years to progress [14].

The occurrence and development of CIA is a complex immune process that is jointly determined by T-cell-induced activation of the immune response, inflammatory factors, production of type II collagen antibody, and other links [14]. This model has typical signs of arthritis, and usually the ankle joint of the hind foot is the most frequently affected.

After immunization, joint involvement can occur as early as the 15th day in rats. Signs of early arthritis can be observed with the naked eye at 21–28 days and include red and swollen joints, which can last for 5–8 weeks in general. We chose 23 days and 40 days as observation time points for early and late arthritis, respectively. The ankle joints of some rats that scored 0 at 23 days had microinflammation, but there was no obvious macroscopic evidence to prove the development of arthritis. In these rats, mild uptake of the imaging agent could be seen in the ankle joint with ^68^Ga-citrate PET/CT, but little uptake was demonstrated ^18^F-FDG PET/CT. Therefore, the *T*/NT ratio of rats with scores of 0 was higher than that of normal control rats. However, on the 40th day, there was only one rat with a score of 0. We think that this rat had not successfully developed arthritis. It is also understandable that the *T*/NT ratio of this rat was lower than that of normal control rats due to data scarcity for calculation. From 23 days to 40 days, although clinical arthritis gradually deteriorated, acute inflammatory manifestations, such as redness, swelling, and increased skin temperature, gradually decreased, and the macroscopic manifestations of arthritis were mainly joint rigidity and deformity. This situation was reflected in the uptake of ^68^Ga-citrate and ^18^F-FDG. The uptake of rats with scores of 0–4 at 23 days was higher than that of rats with a score of 40 days. However, the two PET/CT imaging results showed that the uptake of rats with scores of 3 was lower than that of rats with scores of 2. We tried to explain this finding by HE staining; unfortunately, we do not have a reasonable explanation for this finding. Further experiments are necessary to analyze the reasons for this finding. However, the uptake of ^68^Ga-citrate was significantly higher than that of ^18^F-FDG in arthritic rats with each score, at both 23 and 40 days. Therefore, we think that ^68^Ga-citrate is more valuable for arthritis imaging than ^18^F-FDG. We recommend the use of ^68^Ga-citrate PET/CT in the diagnosis of early RA, which may provide meaningful help for the early clinical treatment of patients.

A report has studied the distribution data of ^68^Ga-citrate in normal rats at different time points (15 min, 30 min, 45 min, 60 min, and 120 min). In that study, blood always showed high uptake, which is consistent with our research results. It is possible that the high uptake is caused by the abundant transferrin in blood. However, the trend of uptake of some organs in that study is inconsistent with our research data. The reason for this difference may be that CIA is a systemic immune disease that may cause damage to multiple organs or tissues. According to the severity of arthritis, the degree of damage to each organ is also different. The ankle joint tissues of the hind feet that we used for determination included bone and muscle tissue, and some of the bony tissue may not have been eroded by inflammatory cells, which may have decreased the %ID/g of the final arthritis tissue samples.

The pathology of the ankle joint observed in CIA model rats is similar to that observed in RA. Neovascularization in the synovium can be observed in the early stages [15]. Abundant blood flow may be the main reason for the increased uptake of ^68^Ga-citrate. These abundant blood vessels supply oxygen to the synovium and release inflammatory factors, causing the synovial tissue to begin to show the characteristics of hyperplasia and hypertrophy, eventually leading to the formation of pannus, erosion of the synovium, and damage to the cartilage and bone [13]. In the later stages of the disease, HE staining showed that there was fibrous tissue and exudative inflammatory cells in the synovial tissue. The medullary cavity of the joint disappeared, the cartilage surface collapsed, a large amount of granulation tissue and fibrous connective tissue filled in, and the bone and cartilage were damaged to varying degrees.

There are some limitations in this study. First, we have a small number of CIA samples; only 10 rats successfully developed arthritis. And the size of the micro-PET/CT acquisition matrix used at our institution was only 128 × 128, which made it impossible to acquire data on the forelimb joints of rats or to semiquantitatively evaluate metabolic changes in the internal organs of rats. Thus, the data from arthritic rats semiquantitatively analyzed by PET/CT may deviate from the real situation. Second, our research results support our recommendation to use ^68^Ga-citrate PET/CT to detect human RA. However, due to the high cost of PET/CT, this examination may be difficult to promote in some institutions that are not equipped with PET/CT.

## 5. Conclusion

In summary, in this study, ^68^Ga-citrate PET/CT was shown to reflect the inflammatory activity of affected joints in CIA rats earlier and more sensitively than ^18^F-FDG PET/CT, and this imaging advantage continues to the late stage of inflammation. ^68^Ga-citrate PET/CT may be helpful in detecting early RA, enabling RA patients to receive early clinical intervention, relieving their clinical symptoms, and reducing the disability rate. This examination method is worthy of clinical promotion and use.

## Figures and Tables

**Table 1:** The *T*/NT of ^68^Ga-citrate and ^18^F-FDG in CIA rats.

	*N*	Day 23	Day 40
^68^Ga-citrate	22	6.34 ± 2.70	5.14 ± 2.10
^18^F-FDG	22	4.71 ± 1.77	3.15 ± 1.26
*t*		6.795	7.819
*P*		<0.001	<0.001

## Data Availability

The data of this study are already presented in this paper.

## Abbreviations

RA: Rheumatoid arthritis
CIA: Type II collagen-induced arthritis
^18^F-FDG: 2-Deoxy-2-[18F]-fluoro-D-glucose
PET/CT: Positron emission tomography/computed tomography
SUV_max_: Maximum standardized uptake value
ROI: Region of interest
HE: Hematoxylin and eosin staining
AI: Arthritis Index
MRI: Magnetic resonance imaging.

## References

[1] Epstein F. H., Harris E. D. (1990). Rheumatoid arthritis. Pathophysiology and implications for therapy. *New England Journal of Medicine*.

[2] Arnett F. C., Edworthy S. M., Bloch D. A. (1988). The American Rheumatism Association 1987 revised criteria for the classification of rheumatoid arthritis. *Arthritis & Rheumatism*.

[3] Boutry N., Morel M., Flipo R.-M., Demondion X., Cotten A. (2007). Early rheumatoid arthritis: a review of MRI and sonographic findings. *American Journal of Roentgenology*.

[4] Bruyn G. A. W., Hanova P., Iagnocco A. (2014). Ultrasound definition of tendon damage in patients with rheumatoid arthritis. Results of a OMERACT consensus-based ultrasound score focussing on the diagnostic reliability. *Annals of the Rheumatic Diseases*.

[5] Rennen H. J. J. M., Boerman O. C., Oyen W. J. G., Corstens F. H. M. (2001). Imaging infection/inflammation in the new millennium. *European Journal of Nuclear Medicine*.

[6] Kubota K., Yamashita H., Mimori A. (2017). Clinical value of FDG-PET/CT for the evaluation of rheumatic diseases: rheumatoid arthritis, polymyalgia rheumatica, and relapsing polychondritis. *Seminars in Nuclear Medicine*.

[7] Beckers C., Ribbens C., Andre B. (2004). Assessment of disease activity in rheumatoid arthritis with (18)F-FDG PET. *Journal of Nuclear Medicine: Official Publication, Society of Nuclear Medicine*.

[8] Burleson R. L., Johnson M. C., Head H. (1973). Scintigraphic demonstration of experimental abscesses with intravenous ^67^Ga citrate and ^67^Ga labeled blood leukocytes. *Annals of Surgery*.

[9] Mirzaei A., Jalilian A. R., Akhlaghi M., Beiki D. (2016). Production of ^68^Ga-citrate based on a SnO_2_ generator for short-term turpentine oil-induced inflammation imaging in rats. *Current Radiopharmaceuticals*.

[10] Chung S.-J., Yoon H.-J., Youn H. (2018). ^18^F-FEDAC as a targeting agent for activated macrophages in DBA/1 mice with collagen-induced arthritis: comparison with ^18^F-FDG. *Journal of Nuclear Medicine*.

[11] Trentham D. E., Townes A. S., Kang A. H. (1977). Autoimmunity to type II collagen an experimental model of arthritis. *Journal of Experimental Medicine*.

[12] Xi C., Tan L., Sun Y. (2009). A novel recombinant peptide containing only two T-cell tolerance epitopes of chicken type II collagen that suppresses collagen-induced arthritis. *Molecular Immunology*.

[13] Lee D. M., Weinblatt M. E. (2001). Rheumatoid arthritis. *The Lancet*.

[14] van den Berg W. B. (2005). Animal models of arthritis. What have we learned?. *Journal of Rheumatology*.

[15] Koch A. E. (2003). Angiogenesis as a target in rheumatoid arthritis. *Annals of the Rheumatic Diseases*.

